# A delayed diagnosis of eosinophilic granulomatosis with polyangiitis complicated with extensive artery occlusion of lower extremities in children: case report and literature review

**DOI:** 10.1186/s12969-019-0331-8

**Published:** 2019-05-28

**Authors:** Xiaoliang Liu, Lin Wang, Kaiyu Zhou, Yimin Hua, Xiaoqing Shi, Chuan Wang

**Affiliations:** 1 Department of Pediatric Cardiology, West China Second University Hospital, Sichuan University, No. 20, 3rd section, South Renmin Road, Chengdu, 610041 China; 2 The Cardiac development and early intervention unit, West China Institute of Women and Children’s Health, West China Second University Hospital, Sichuan University, Chengdu, Sichuan China; 3 Key Laboratory of Birth Defects and Related Diseases of Women and Children (Sichuan University), Ministry of Education Chengdu, Chengdu, Sichuan China; 4 Key Laboratory of Development and Diseases of Women and Children of Sichuan Province, West China Second University Hospital, Sichuan University, Chengdu, Sichuan China; 5 Longquanyi District of Chengdu Maternity & Child Health Care Hospital, Chengdu, Sichuan China

**Keywords:** Delayed diagnosis, Eosinophilic granulomatosis with polyangiitis, Asthma, Arteries occlusion, Children

## Abstract

**Background:**

Eosinophilic granulomatosis with polyangiitis (EGPA) is a rare systemic vasculitis in children. A delayed or missed diagnosis of pediatric EGPA is common, owing to the atypical clinical manifestation and limited recognition of this disorder. The vasculitis in EGPA typically involves small to medium size vessels. Extensive occlusion of arteries in the extremities was being extremely rare and has never been reported in children.

**Case presentation:**

A 10-year and 10-month-old girl with recurrent wheezing and breathlessness during exercise, was initially diagnosed with asthma at the age of five years. Despite unexplained manifestations, including intermittent remarkably increased eosinophilia, uncontrolled allergic rhinitis, and recurrent petechia, from the onset of asthma through to its remission, the consideration of EGPA was completely ignored until the patient presented with aggravated petechia and severe ulceration of the lower extremities, associated with extensive stenosis and/or occlusion of the arteries of the shank and foot. Given her history of asthma, eosinophilia, allergic rhinitis, mononeuropathy, pulmonary infiltrates, and vasculitis confirmed by the skin biopsy, the diagnosis of EGPA was ultimately confirmed. Regrettably, the initial inappropriate and irrational use of corticosteroid failed to relieve the symptoms until more aggressive treatment with intravenous methylprednisolone was started. This was followed by methotrexate treatment, with tapering of prednisone, without relapse over a six-month follow-up.

**Conclusions:**

Pediatric rheumatologists should be alert to the possibility of EGPA in children with refractory asthma associated with unexplained manifestations, and should be aware of the thromboembolic complications as vascular sequelae of EGPA.

## Background

Eosinophilic granulomatosis with polyangiitis (EGPA, formerly Churg-Strauss syndrome) is a rare kind of systemic vasculitis and mainly affects adults [1, 2]. With induction of remission (high-dose corticosteroid alone or combined with another immunosuppressant such as cyclophosphamide), followed by maintenance of remission therapy (low-dose corticosteroids and/or a maintenance immunosuppressive agent such as azathioprine or methotrexate) or introduction of second-line therapy (mycophenolate mofetil, rituximab, plasma exchange) for failed induction, the prognosis of adult EGPA is generally satisfactory [3]. EGPA in children are rarely reported [4] and appears to have a relatively poor prognosis, most possibly resulted from a delayed or missed diagnosis and more rapid progress [5, 6]. Discouragingly, due to the rarity of pediatric EGPA, atypical clinical manifestations at initial stage and particularly limited recognition of this disorder, it is still substantially difficult to establish a timely and accurate diagnosis.

Besides, although the vasculitis in EGPA can involve small and medium-sized muscular arteries, capillaries, veins and venules, the stenosis and blockage of medium-sized arteries is only described in several case reports [7–20]. Artery occlusion of extremities is extremely rare and only five cases have been documented in adults [10, 13–16]. There are currently no similar reports in children. Herein, a delayed diagnosis of EGPA in children for over five years since the asthma symptom onset, complicating with extensive artery occlusion of lower extremities, was firstly reported, aiming to alert pediatric rheumatologists the possibility of EGPA in refractory asthma patients with unexplained manifestations, and improve the recognition of the thromboembolic complications as vascular sequelae in EGPA for clinicians.

## Case presentation

A 10 years and 10 months old girl was admitted into our hospital with a complaint of recurrent wheezing and breathlessness during exercise for several months five years ago. At that time, respiratory tract infection, foreign body aspiration, airway developmental anomalies, cardiovascular diseases, and tumors, assessed by chest computed tomography (CT), CT angiography (CTA), bronchoscopy, and echocardiography, were excluded. The diagnosis of asthma was considered, based on the history of allergic rhinitis, elevated level of serum eosinophil (6.8 × 10^9^/L, reference, 0–0.8 × 10^9^/L), and immunoglobulin E (IgE, 1128 IU/mL; reference: 0–165 IU/mL), a reversible airway obstruction detected by spirometry (resulting in a ≥ 12% increase in the predicted FEV1) and a positive response to the therapy of rapid acting β2-agonist and inhaled glucocorticoids. The child’s adherence to the treatment and adequate performance of the inhalation technique were confirmed. Yet, her asthma was refractory to initial treatment. The symptoms of asthma were gradually brought under control, with the use of double inhaled glucocorticosteroid and leukotriene modifier, over a period of three years, at which time the dosage was gradually decreased. Eight months prior to the current admission, all drugs had been withdrawn, without a relapse of asthma. However, symptoms of allergic rhinitis did not resolve, with intermittent elevation of serum levels of eosinophils persisting. Additionally, the patient complained of recurrent petechia on her lower limbs, occurring on average once per year, over the past three years. Unfortunately, all these signs failed to gain clinicians’ attention and no further examinations were performed.

At this time, she was admitted to our hospital with a one-month history of lower extremity numbness, arthralgia and myalgia of lower limbs with claudication, but without fever, edema, fatigue or weight loss. There was no history of trauma prior to the onset of these symptoms. On admission, the physical examination revealed decreased skin temperature of both lower limbs, swelling of the left calf, subcutaneous nodules and palpable purpura below the knee. Pulse of the dorsal pedis and radial arteries were absolutely absent. No other positive findings were identified. For the auxiliary examination, a peak eosinophilia of 7.33 × 10^9^/L (51.5%, reference: 0–8%) was detected on complete blood analysis. An elevated serum level of IgE (referred to 1710 IU/mL) and erythrocyte sedimentation rate (ESR) of 34 mm/h (reference: 0–26 mm/h) were observed. The possibility of tuberculosis or parasite infection was not considered as there was no history of probable contact, as well as negative results on the BCG, PPD test and T-SPOT, with no specific parasite antibodies identified (*Fascioliasis*, *Clonorchiasis*, *Paragonimiasis*, *Schistosomiasis*, *Toxoplasma*). As well, all other laboratory tests were within normal limits, including urinalysis, hepatic function, renal function, myocardial function, coagulation function, blood gas analysis, serum level of glucose, blood ammonia, lipid panel, C-reaction protein (CRP), autoantibody, antineutrophil cytoplasmic antibodies (ANCAs, including p-ANCA and c-ANCA) and human leucocyte antigen-B27 (HLA-B27). Marrow cytology inspection suggested a proliferation of granulocytes and an elevated percentage of eosinophil, which excluded the possibility of common blood system diseases. Remarkably, extensive occlusion of the anterior tibial and dorsal pedis arteries were identified, bilaterally, using a vascular ultrasound examination. Electromyogram (EMG) revealed a deficit of both motor and sensory fibers of peripheral nerves, suggestive of a peripheral neuropathy. Combined with the significant history of asthma, eosinophilia> 10%, recurrent petechia, peripheral neuropathy, and the vascular findings, the suspicion of EGPA was strongly raised.

**Fig. 1:**
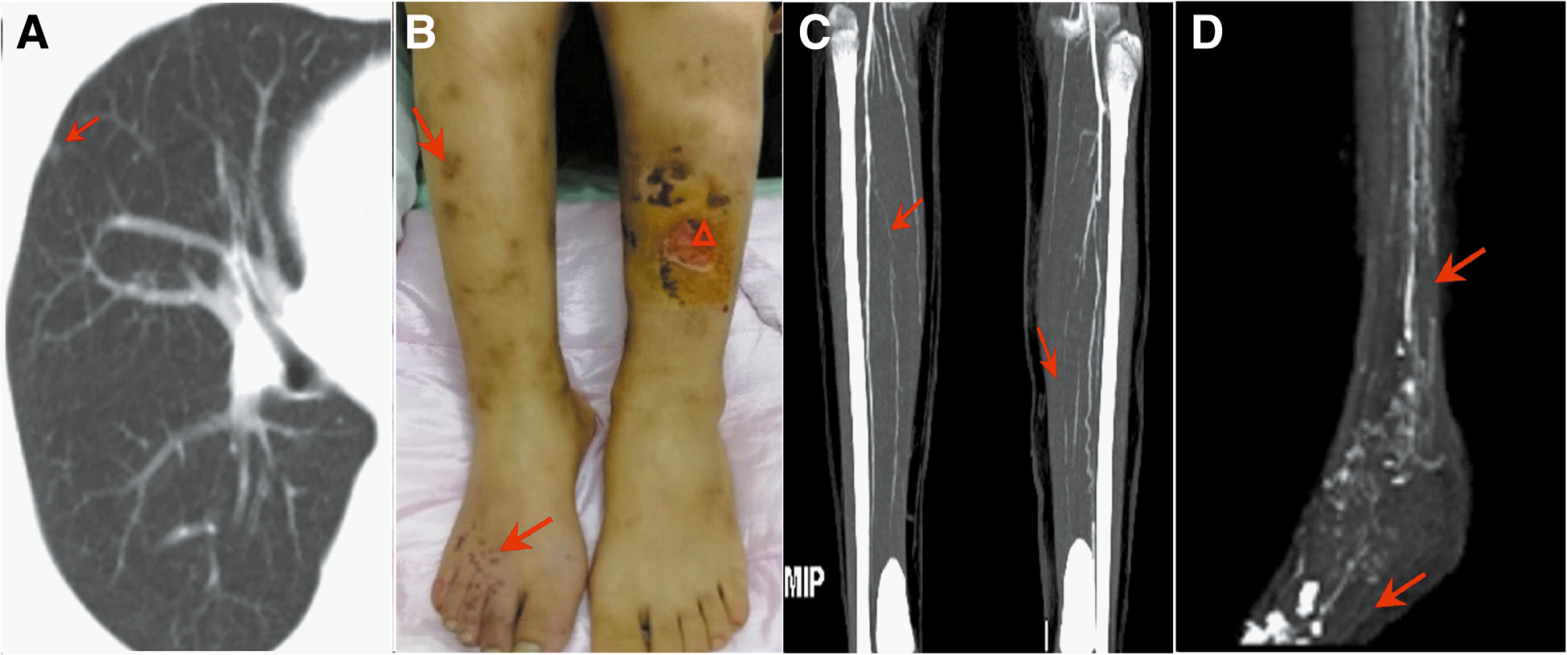
**a**: The chest CT scan showed that a change of ground-glass opacities and nodule was found on the middle lobe of the right lung (red arrow) **b**: Skin lesion presenting as purpura in lower limbs (red arrow), and an ulceration with purulent exudate, sized 3 cm * 4 cm in the left leg (triangle), **c-d**: The CTA of lower extremities showed extensive stenosis and/or occlusion of shank and foot arteries (red arrow)

However, since EGPA rarely occurs in children, more comprehensive examinations were carried out to confirm our hypothesis. A change of ground-glass opacities and nodule was found on the middle lobe of the right lung on chest CT imaging (Fig. 1a). The skin biopsy, obtained from a petechia on the patient’s left lower extremity, demonstrated eosinophilic infiltration and necrotizing vasculitis, characterized by neutrophils and mixed lymphocyte infiltration, striking fibrinoid necrosis, endothelial and muscle cell necrosis in peri-vascularly and the dermis, but absence of granuloma formation. Based on the 1984 Lanham criteria [21] and the 1990 American College of Rheumatology (ACR) criteria [22], the diagnosis of EGPA was ultimately established. There was no evidence of ear, nose and throat (ENT), ocular, gastrointestinal, and cardiac manifestations. Hydrocortisone (10 mg/kg/d) treatment was initiated for a period of five days, subsequently followed by oral prednisone (1 mg/kg/d). The treatment was sufficient to achieve partial control of her status.

Unfortunately, five days later, the purpura attacked her lower limbs, as well as an ulceration with purulent exudate, sized 3 cm*4 cm in the left leg (Fig. 1b). She was treated with the antibiotics and debridement for a period of 3 days, but with any remission. New findings were observed on vascular ultrasound, namely, occlusion of the distal portion and of the posterior tibial arteries, and thrombo-arteritis of the radial and ulnar arteries. Systemic CTA identified extensive stenosis and/or occlusion of shank and foot arteries with formation of collateral vessels (Fig. 1c-d). For our low recognition and poor awareness of EGPA in children, we failed to perform a comprehensive evaluation for vasculitis and provide the patient with an inadequate therapy of corticosteroid (hydrocortisone) during her initial period of hospitalization. After the overall evaluation of her condition, intravenous methylprednisolone (15 mg/kg/d) was initiated for three days, and then a sequential therapy of oral prednisone (2 mg/kg/d) was continued. Antithrombotic treatment (clopidogrel: 25 mg daily, intracutaneous injection of nadroparin: 88.5 IU /Kg q12h, phentolamine: 5 μg/kg/min) was also initiated. The administration of oral methotrexate (12.5 mg per week) was started on day 7 after the end of the methylprednisolone treatment. Ultimately, control of the vasculitis was achieved and the patient was discharged home after the purpura gradually disappeared and the ulcer healed, with a return to normal levels of peripheral eosinophil count and ESR. Oral medication was prescribed, consisting of prednisone, methotrexate, clopidogrel, folic acid, and beraprost sodium. After a six-month period of stability in the disease status, methotrexate was continued, with tapering of the prednisone, without relapse.

## Discussion

In children, EGPA is a quite rare form of vasculitis. The three largest pediatric EGPA series merely reported 9 [23], 13 [5] and 14 [6] cases, respectively. Typically, EGPA is described as a pathological triad, evolving from a prodromal phase characterized by asthma, ENT involvement including nasal polyps, allergic rhinitis, recurrent or chronic sinusitis, and secretive otitis media, chronic ear drainage, sensorineural hearing loss, facial nerve palsy, to an eosinophilic phase of peripheral blood eosinophilia and eosinophilic infiltration of multiple organs, especially the lung, cardiac and gastrointestinal tract, and ending in the final phase of systemic vasculitis mainly manifested with peripheral neuropathy, renal involvement and skin lesions. The symptoms of fever, elevated serum levels of CRP and ESR were not specific signs of EGPA and are not essential according to the diagnostic criteria for EGPA [24, 25]. Due to our limited recognition and poor awareness of this disorder, despite with classic clinical features and process of EGPA, the diagnosis of EGPA for our patient was delayed for over five years, which was longer than previously reported in the literature [5, 6, 23]. In fact, a previous report indicated that most cases of pediatric EGPA were diagnosed during the eosinophilic phase [6], whereas our patient had evolved to the phase of systemic vasculitis. Moreover, remission was not achieved in our patient owing to an inadequate induction therapy of corticosteroids, possibly leading to the rapid progression of the vasculitis. Hence, our report of this rare case of delayed EGPA diagnosis and the resulting thromboembolic complications as vascular sequelae is clinically important. Based on our experience, we strongly suggest that pediatricians, particularly pediatric rheumatologists, should be alert to the possibility of EGPA in children presenting with refractory asthma, especially those with unexplained manifestations. Clinically, enhanced awareness of the vascular sequelae in EGPA could improve outcomes for these children as the prognosis may benefit from prompt, adequate and even more aggressive treatment.

Overall, pediatric EGPA presents much worse outcomes with higher rate of relapse and mortality than adults [5, 23, 26], partly attributing to the delayed diagnosis and more rapid evolution of the disease in children than adults. Therefore, establishing a timely diagnosis of EGPA in children is critical important, and yet this diagnosis remains challenging as the diagnostic criteria of EGPA in children have been formulated based on the adult presentation of EGPA. From our experience, and after a review of the literature [2, 5, 6, 23, 24], there were some signs that could have led us to an earlier diagnosis of EGPA in our pediatric patients. Firstly, as a major diagnostic criterion, hypereosinophilia was generally higher than 1.5 × 10^9^/L, with a level as a high as high as 51.6 × 10^9^/L having previously been reported [27]. Almost all patients in the three largest pediatric EGPA case series [5, 6, 23] showed a marked hypereosinophilia, with a median level across these three case series reports of 7.01 × 10^9^/L (0.52–14.4), 6.2 × 10^9^/L (2.35–22.55) and 9.34 × 10^9^/L (3.25–13.42), respectively. Although extrinsic asthma is often accompanied by eosinophilia, the increased level in asthma seldomly exceeds 0.8 × 10^9^/L [28]. Therefore, the possibility of EGPA in children should be alerted with refractory asthma and remarkable hypereosinophilia after excluding other causes. Secondly, EGPA can involve multiple organs and tissues, including skin lesions, renal involvement, mono or polyneuropathy, pulmonary infiltrates, and allergic rhinitis. Therefore, a diagnosis of EGPA would be reasonable to investigate in patients with asthma presenting with involvement of other organs and tissues.

The vasculitis in EGPA can involve small and medium-sized muscular arteries, capillaries, veins and venules. However, as shown in Table 1, an extensive occlusion of arteries in the extremities has only reported in five adult patients [10, 13–16], and not in children. Although the risk factors for artery occlusion in EGPA has not been clearly elucidated, it has been shown that EGPA combined with negative p-ANCA ANCA is associated with a much higher tissue infiltration by eosinophils [29], which substantially increases the risk for thrombosis [16, 20]. Notably, all five cases of EGPA-related arterial occlusion in the extremities listed in Table 1, as well as the patient in our case report, tested negative for ANCAs. In addition, the inappropriate and irrational use of corticosteroids might fail to achieve symptom relief and disease remission, but inversely worsen the conditions by contributing to hypercoagulability and thrombosis formation.

**Table 1:** Summary of reported EGPA patients with occlusion of arteries in the extremities

Author, year	Braunberger T, 2016	Waseda K, 2011	Otani Y, 2003	Inui S, 2001	Abbas MA, 2000	Present study, 2018
Country	USA	Japan	Japan	Japan	UK	China
Sex	Male	Female	Male	Male	Female	Female
Age, years	72	37	59	69	49	10.8
Clinical manifestations						
Asthma	+^a^	+	+	+	+	+
Eosinophilia	-^b^	+	+	+	+	+
Neuropathy	+	–	+	+	+	+
Pulmonary	–	–	–	–	+	+
Skin lesions	+	–	+	+	–	+
Extrapulmonary	Weight loss, fever	Fever	Liver infarction	Atrial fibrillation, fatigue, fever, weight loss	Microhaematuria, proteinuria, arthralgia,	Arthralgia and myalgia
Peak level of eosinophils	Normal	1.9 × 10^9^/L	12.9 × 10^9^/L	15.8 × 10^9^/L	13.1 × 10^9^/L	7.3 × 10^9^/L
ANCAs	Negative	Negative	Negative	Negative	Negative	Negative
Presentation of vasculitis	Occluded right anterior tibial artery	Narrowing of the dorsalis pedis artery and of the peripheral arteries of her right leg	Narrowing of the main arteries in the forearms and lower legs; narrowing and occlusion of the hepatic vein	Stenosis of the medium sized arteries in both legs	Digital vasculitis affecting the left index and middle finger	Extensive stenosis and/or occlusion of shank and foot arteries with formation of collateral vessels
Treatment	CTX, methylprednisolone, maintaining with prednisone	Prednisolone, CSA, CTX, Antithrombotic agents, azathioprine	Prednisolone, intravenous PG1	Prednisolone	Methylprednisolone, CTX	Methylprednisolone maintain with prednisone, methotrexate, antithrombotic agents
Prognosis	Right partial second toe amputation, alive	Plastic surgery, alive	Amputate, alive	Alive	Alive	Alive

Abbreviations: *ANCAs* antineutrophil cytoplasmic antibodies, including p-ANCA and c-ANCA, *CSA* cyclosporin A, *CTX* cyclophosphamide, *PG1* prostaglandin E1

^a^Positive, ^b^Negative

## Conclusions

EGPA is a substantial rarity of vasculitis in children. Recognition and awareness of this disorder is extremely limited. Our present case emphasizes the importance of considering the diagnosis of EGPA in children presenting with refractory asthma and unexplained manifestations. It is important to note the possible development of extensive occlusion of arteries in the extremities in pediatric patients with EGPA. Delayed diagnosis may worsen the prognosis and even lead to a fatal outcome. Therefore, timely and proper treatment would be important to achieve remission of the vasculitis process.

## Data Availability

All data are included in this published article.

## Abbreviations

ANCAs: Antineutrophil cytoplasmic antibodies
BCG: Bacillus Calmette-Guerin
CRP: C-reaction protein
CT: Computed tomography
CTA: CT angiography
EGPA: Eosinophilic granulomatosis with polyangiitis
EMG: Electromyogram
ENT: Ear, nose and throat
ESR: Erythrocyte sedimentation rate
FEV1: The forced expiratory volume in 1 s
HLA-B27: Human leucocyte antigen-B27
IgE: Immunoglobulin E
